# Editorial: Experimental Approaches to Pragmatics

**DOI:** 10.3389/fpsyg.2022.865737

**Published:** 2022-04-19

**Authors:** Valentina Cuccio, Pietro Perconti, Gerard Steen, Yury Shtyrov, Yan Huang

**Affiliations:** ^1^Department of Ancient and Modern Civilizations, University of Messina, Messina, Italy; ^2^Department of Cognitive Sciences, Psychology, Education and Cultural Studies, University of Messina, Messina, Italy; ^3^Language and Communication, Faculty of Humanities, University of Amsterdam, Amsterdam, Netherlands; ^4^Center for Functionally Integrative Neuroscience, Institute for Clinical Medicine, Aarhus University, Aarhus, Denmark; ^5^Faculty of Arts, The University of Auckland, Auckland, New Zealand

**Keywords:** experimental pragmatics, neuropragmatics, context, clinical pragmatics, figurative language

## Introduction

Often the starting point of the study of the biological bases of language is the question: How is language represented in the brain? This question might suggest that linguistic meaning is a form of knowledge stored in the human brain that we retrieve when we listen to or read words and sentences. Undoubtedly, we do have a knowledge of language which is represented in neural networks in the perisylvian cortex. However, the way this question is formulated can be misleading. A better way to rephrase it would be: how meaning is constructed in the brain? The difference between the first and the second question is the model of language they imply. The first question presupposes that language is a code whereas the second suggests that linguistic meaning is always the result of a contextually based process of interpretation. Many linguistic phenomena, such as metaphors, irony and other forms of figurative meaning, could hardly be explained if we defined language as a code. A pragmatic approach is, thus, fundamental if we aim at providing a full account of language processing. We need to explain how we use symbols and how we make meanings out of them. And we need to do so in a psychologically and neurologically plausible framework.

Since the pioneering work of Wittgenstein and Grice, Pragmatics, the study of how language is used in context, has been traditionally addressed by philosophers and linguists from a theoretical perspective. However, classic pragmatic notions such as communicative intentions, implicatures or usage-based meaning must now be understood in light of a psychological and neural account of language. Thus, today, Pragmatics is a highly interdisciplinary enterprise that is investigated by psychologists, neuropsychologists and neuroscientists as well as philosophers and linguists. Recently, this experimental approach to the pragmatics of language has gathered momentum and has given rise to the birth of a new field of study: Experimental Pragmatics (Cuccio, 2022; Gibbs and Colston for an overview). This refers to a set of different but strictly interrelated disciplines: *Neuropragmatics*, which aims at identifying the neural infrastructures underlying pragmatic processes in language production/comprehension; *Clinical Pragmatics*, which aims at studying pragmatic disorders in clinical populations and *Experimental Pragmatics stricto sensu*, which aims at empirically validate theoretical accounts of the pragmatic of language by means of behavioral experiments.

With no ambition to provide the precise geography of this research field, we can say that *Experimental Pragmatics stricto sensu* is, no doubt, the discipline most represented in this collection. Thirteen papers out of eighteen accepted for publication in the Research Topic *Experimental approaches to Pragmatics* investigated crucial theoretical issues in the pragmatics of Language by means of behavioral studies and surveys. Two theoretical contributions (Gibbs and Colston; Rizzato) provided a critical overview of current perspectives in the experimental approaches to pragmatics with a specific focus on metaphors. One paper (Schaeken et al.), investigating the role of working memory in the processing of scalar implicature in Schizophrenic patients, was framed within the discipline of *Clinical Pragmatics*. Two papers (Brilmayer and Schumaker; Spychalska et al.) used electrophysiological measures in two Event Related Potential (ERP) experiments to investigate, respectively, scalar implicature in full and partial information context and the relationship between referential chains and predictive processes. These ERP studies were carried out in the framework of *Neuropragmatics*. The latter seems to be still the most underrepresented branch of investigation in the study of the neural correlates of language.

## Pragmatics and the Neural Correlates of Language

### A Place for the Pragmatics of Language

Experimental pragmatics is today a burgeoning field of study with a very lively debate (Gibbs and Colston). However, the research about the brain areas underpinning the pragmatic processing involved in non-literal usages of language (i.e., Neuropragmatics; see Bambini, 2010; Bara and Bara, 2010; Haggort and Levinson, 2014) is relatively recent compared to the study of the neural correlates of syntax and semantics.

To understand the reasons of this gap we need to acknowledge that in the second half of the 19° century and for a long time in the Neuroscience of language, Pragmatics was not even considered as one of the levels, along with phonology/orthography, syntax and semantics, to be taken into consideration when exploring the neural correlates of language. Linguists did not recognize the pragmatic dimension of language until the work of philosophers of language such as Wittgenstein and Anscombe (1953), Austin and Urmson (1962), and Grice (1989). And, when Pragmatics was finally introduced in the theoretical study of language, in the second half of the 20° century, it was first considered as a far less important feature compared to syntax and semantics. In fact, as Mey (2001) clearly explains in his introduction to Pragmatics, it was first considered as the “waste-basket of semantics,” a place where linguists were used to relegate problematic aspects of language, such as its figurative usages, which they could hardly explain in semantic theories. Thus, Pragmatics struggled to find its identity and its own place in Linguistics (Mey, 2001). And if linguists for a long time did not sufficiently consider Pragmatics, so did, later, the neuroscientists working on the identification of the anatomical bases of language. For this reason, Neuropragmatics, compared to the study of the neurobiology of syntax and semantics, is the most recent branch of Neurolinguistics.

Furthermore, the possibilities of experimental pragmatics have long been undermined by the difficulties of modeling context dependence in an adequate way. Formal semantics has made many important contributions by attempting to bring to light the logic underlying the fact that the meaning of words and sentences often seems to depend on the context of production and evaluation of the linguistic act we are considering. On the whole, however, the adventure of formal semantics and, in particular, the attempt to provide a satisfying logical model of the phenomenon of context dependence has not been a success. The problem is that, if we are without a logical model of context dependence, quantitative research has a poor basis, and with it the very possibilities of doing experimental research in the field of pragmatics.

### From Aphasiology to the Contemporary Cognitive Neuroscience of Language

The beginning of the identification of the neural structures subserving language dates back to the second half of the 19° century, when the development of aphasiology made possible the first description of the brain areas underlying the processing of language. Broca (1861) discovered that language is lateralized to the left hemisphere and identified a region in the frontal lobe, the pars triangularis of the Inferior Frontal Gyrus (IFG—Brodmann area 45) which seemed to be responsible for language production. A few years later, Wernicke (1874) identified in the temporal lobe another area linked to language, the Superior Temporal Gyrus (STG—Brodmann area 22), which, in turn, seemed to be related to the comprehension of language. On these bases, Wernicke (1874) proposed a first model of the brain mechanisms underlying both language production and comprehension, which was then further developed by Lichtheim (1885) and, in the second half of the 20° century, renewed by Norman Geschwind. The Wernicke-Lichtheim model, also known as the Wernicke-Geschwind model, for the processing of language has been influential for a long time. Generally speaking, aphasiology certainly gave a fundamental impulse to the study of the brain bases of language. However, today models of language processing based on aphasiology have been largely revised (for a discussion, Kandel et al., 2013). Recent years have witnessed an enormous technological growth. Functional brain imaging research allowed us to study *in vivo* the brain of both healthy subjects and patients with language impairments while these perform linguistic tasks. Techniques such as the functional Magnetic Resonance Imaging (fMRI) or the magnetoencephalography (MEG) provided us with the possibility to observe the neural mechanisms underlying the processing of language. On these bases, more complex models of the functional neuroanatomy of language have been proposed (e.g., Hickok and Poeppel, 2004, 2007; Friederici and Gierhan, 2013). Today we know that several systems underpin the processing of language and that the language network is far more sophisticated and extended than it was first believed. Broca's and Wernicke' area are still considered as the cornerstone of this network but they are functionally characterized in a partially different way. In fact, these brain areas not only subserve production and comprehension of language, as it was first believed on the basis of neurological data. In the field of language processing, they are today mainly characterized in terms of their involvement, respectively, in the processing of syntax and semantics. Furthermore, we know that the arcuate fasciculus, that links Broca and Wernicke areas, previously considered to be unidirectional, conveying information from Wernicke to Broca, is bidirectionally linked to these two brain regions (Hickok and Poeppel, 2004, 2007). Most importantly, Broca and Wernicke areas are also connected through two other streams of information, beyond the arcuate fasciculus: (i) a ventral stream, bilaterally distributed in the brain, has been identified in the superior and middle temporal lobes, although with some differences in the recruitment of the left and right hemispheres. This ventral stream processes speech signals for language comprehension (i.e., it maps sounds to meanings, according to Hickok and Poeppel, 2004, 2007 model); (ii) a dorsal stream, lateralized to the left hemisphere, includes structures in the posterior frontal lobe and in the posterior-dorsal area of the temporal lobe. This dorsal stream maps acoustic representations of language to articulatory networks (i.e., it maps sounds to articulatory gestures; for a discussion of the model, Hickok and Poeppel, 2004, 2007). In addition to this, other regions linked to language processing have also been identified in the perisylvian cortex (see Kandel et al., 2013 for anatomical and functional description of these areas). Furthermore, techniques with high temporal resolution, such as, for example, the MEG or the electroencephalography (EEG), also gave us the possibility to investigate the neural time course of language processing, which is of paramount importance to develop a model of how we produce and comprehend language since language production/comprehension is a multilayered process where different kinds of information need to be handled.

Thanks to this enormous technological growth and, most of all, to the introduction of more fine-grained models of language use, today we know that the neural network recruited by the processing of context-based meaning is bilaterally represented in the brain and it includes regions such the Inferior Frontal Gyrus (IFG; left and right BA 45, left BA 47), the Temporal-Parietal Junction (TPJ, right and left BAs 22 and 39), the right Anterior Cingulate Cortex (ACC, right BAs 24 and 32) and the right dorsolateral Prefrontal Cortex (DLPFC), specifically the middle frontal gyrus (MFG, right BA 9).

Many questions are open in the experimental study of the pragmatics of Language. Experimental research in this research field is intense today. To understand the psychological and neural processes subserving our ability to use language in context is of paramount importance for a better comprehension of many clinical conditions and, most of all, for a deeper understanding of what makes us human.

## Author Contributions

Although all the authors discussed and designed the article together, Sections Introduction and From Aphasiology to the Contemporary Cognitive Neuroscience of Language were written by VC. Section A Place for the Pragmatics of Language was written by VC and PP. All authors contributed to the article and approved the submitted version.

## Conflict of Interest

The authors declare that the research was conducted in the absence of any commercial or financial relationships that could be construed as a potential conflict of interest.

## Publisher's Note

All claims expressed in this article are solely those of the authors and do not necessarily represent those of their affiliated organizations, or those of the publisher, the editors and the reviewers. Any product that may be evaluated in this article, or claim that may be made by its manufacturer, is not guaranteed or endorsed by the publisher.
